# Caddisfly Silk-Polycaprolactone Foams: Physicochemical and Biological Properties of Nature-Inspired Biomaterials

**DOI:** 10.3390/jfb16060199

**Published:** 2025-05-29

**Authors:** Mateusz M. Urbaniak, Mariusz Tszydel, Konrad Szustakiewicz, Aleksandra Szwed-Georgiou, Bartłomiej Kryszak, Marcin Włodarczyk, Sylwia Michlewska, Piotr Jóźwiak, Tomislav Ivankovic, Mikołaj K. Cybulski, Karolina Rudnicka

**Affiliations:** 1 Department of Immunology and Infectious Biology, Faculty of Biology and Environmental Protection, University of Lodz, 12/16 Banacha, 90-237 Lodz, Poland; mateusz.urbaniak@edu.uni.lodz.pl (M.M.U.); aleksandra.szwed@biol.uni.lodz.pl (A.S.-G.); marcin.wlodarczyk@biol.uni.lodz.pl (M.W.); 2 Department of Inorganic and Analytical Chemistry, Faculty of Chemistry, University of Lodz, 12 Tamka, 91-403 Lodz, Poland; 3 Department of Ecology and Vertebrate Zoology, Faculty of Biology and Environmental Protection, University of Lodz, 12/16 Banacha, 90-237 Lodz, Poland; mariusz.tszydel@biol.uni.lodz.pl; 4 Department of Polymer Engineering and Technology, Faculty of Chemistry, Wroclaw University of Science and Technology, 27 Wyb. Wyspianskiego, 50-370 Wroclaw, Poland; konrad.szustakiewicz@pwr.edu.pl (K.S.); bartlomiej.kryszak@pwr.edu.pl (B.K.); 5 Laboratory of Microscopic Imaging and Specialized Biological Techniques, Faculty of Biology and Environmental Protection, 12/16 Banacha, 90-237 Lodz, Poland; sylwia.michlewska@biol.uni.lodz.pl; 6 Department of Invertebrate Zoology and Hydrobiology, Faculty of Biology and Environmental Protection, University of Lodz, 12/16 Banacha, 90-232 Lodz, Poland; piotr.jozwiak@biol.uni.lodz.pl; 7 Division of Microbiology, Faculty of Science, Department of Biology, University of Zagreb, Ravnice 48, 10000 Zagreb, Croatia; tomislav.ivankovic@biol.pmf.hr; 8 Institute of Human Biology and Evolution, Faculty of Biology, Adam Mickiewicz University, 6 Uniwersytetu Poznanskiego, 61-614 Poznan, Poland; mikcyb@amu.edu.pl

**Keywords:** polycaprolactone, caddisfly, silk, tissue regeneration, nature-inspired biomaterials

## Abstract

The unique properties of insect silk have attracted attention for years to develop scaffolds for tissue engineering. Combining natural silks with synthetic polymers may benefit biocompatibility, mechanical strength, and elasticity. Silk-modified biomaterials are a promising choice for tissue engineering due to their versatility, biocompatibility, and many processing methods. This study investigated the physicochemical and biological properties of biocomposites formed by combining caddisfly silk (*Hydropsyche angustipennis*) and polycaprolactone (PCL). The PCL foams modified with caddisfly silk demonstrated full cytocompatibility and enhanced fibroblast adhesion and proliferation compared to unmodified PCL. These silk-modified PCL foams also induced NF-κB signaling, which is crucial for initiating tissue regeneration. Notably, the antimicrobial properties of the silk-modified PCL foams remained consistent with those of unmodified PCL, suggesting that the addition of silk did not alter this aspect of performance. The findings suggest that caddisfly silk-modified PCL foams present a promising solution for future medical and dental applications, emphasizing the potential of alternative silk sources in tissue engineering.

## 1. Introduction

The high morbidity and increasing frequency of degenerative diseases in humans and animals force the development of advanced and functional prostheses, dressings, or active intrabody structures [1,2]. Ongoing research aiming to develop novel biomaterials for tissue engineering includes synthetic and bio-inspired polymers, despite the various limitations of each. In the first case, an easy production process and its modifications are advantageous, while in the second one, it has higher cyto- and biocompatibility [3,4]. All mentioned features are combined in hybrid materials, simultaneously creating new properties for the resulting biomaterials [5], supporting their directional action in saturation with various natural compounds [6,7,8] or metal oxides [9,10,11]. Nowadays, when creating new materials, attention is paid to not only that they should be indifferent to the recipient’s organism but also active and biodegradable during the treatment process [12]. In recent years, the natural polymer silk fibroin (SF) has become a subject of interest in tissue engineering due to its good biocompatibility and relatively high bioactivity. Therefore, this material has been broadly used in musculoskeletal engineering for tissue regeneration [13,14,15]. The first patent for a silk-based porous scaffold produced from a freeze-dried native silk solution (i.e., silk extracted directly from the silk gland of the mulberry silkworm) was issued in 1987 [16]. Biomaterials that are hybrids of silkworm fibroin with calcium phosphate, graphene oxide, titanium dioxide, silica, bioactive glass, silver, polycaprolactone, collagen and gelatin, chitosan, extracellular matrix, cellulose, or alginate have already been developed for sports medicine [17]. Multicomponent hybrids are also possible, especially since biphasic and multiphase scaffolds can be more effective in repairing interface tissue than single-phase scaffolds [18,19,20,21]. The major disadvantages of SF scaffolds include the low cell-adhesive activity and difficulty in the independent tuning of mechanical and biodegradation properties [22,23]. Silk derived from the mulberry silkworm must also be modified for medical use, which may result in detrimental changes in the mechanical properties [24]. In addition, moisture inside the human body constitutes a potential challenge since it softens and weakens silk produced by terrestrial arthropods.

Thousands of invertebrate species spin silk, and the amino acid composition and properties of silk fibroin from various taxa can differ from those of widely used silk produced by *Bombyx mori* [25,26,27]. Silk produced by caddisfly larvae is an interesting and innovative modifier of biomaterials that can be used for medical purposes. As in closely related butterflies, silk is spun by spinnerets located near the mouth [28]. Silk secretion is the final product of paired silk glands, which are modified salivary glands. The proteins biosynthesized in the trichopteran silk glands are the silk thread’s precursors as well as for the underwater cement (depending on the taxonomic group of caddisflies). The formation of silk threads or filling mass is regulated in the silk glands by phosphate residues binding cations of divalent elements. Multivalent metal cations (mainly Ca^2+^ and Mg^2+^) are integrated into the silk fibers from the environment during the spinning process rather than being incorporated into the silk precursors in the silk gland. The silk fibroin produced by the Trichoptera is similar to Lepidoptera and consists of homologues of both H-fibroin and L-fibroin; however, it lacks glycoprotein P25 as well as sericin encasement bonding the double strand [29,30]. From a commercial point of view, the use of trichopteran silk is advantageous due to its high charge density, which is acquired through the phosphorylation of serines and the accumulation of basic residues [31]. Post-translational phosphorylation of serine provides a multitude of potential interfacial adhesion mechanisms [30,32]. Firm adhesion is likely due to covalent cross-linking and electrostatic interactions [33,34]. Phosphorylation of serines that are also found in their terrestrial cousins—butterflies and moths—may be primarily responsible for the adaptation of the ancestral dry silk to the underwater environment of caddisfly larvae [31]. In trichopteran silk, phosphate groups are accompanied by a high proportion of basic residues, which are nearly absent in terrestrial silks [35]. As a result, caddisfly H-fibroins are polyampholytic [31]. Phosphates can form several types of water-resistant bonds, which makes them highly effective and versatile adhesion promoters in wet conditions [30,32].

Currently, obtaining silk from caddisfly larvae in industrial quantities is as feasible as acquiring silk from spiders [36]. Therefore, we tested the unique properties of the silk secretion from the *Hydropsyche angustipennis* species as an extender for a synergistic combination with an artificial polymer. Following the suggestions of Cai et al. [37], He et al. [38], and Wang et al. [39] a biocomposites were created as a scaffold for the following examinations. These studies aimed to determine the physicochemical and biological properties with an emphasis on potential medical or dental applications.

The aim of the present study was to verify the feasibility of developing a composite material based on polycaprolactone (PCL) modified with caddisfly silk and to evaluate its cytocompatibility, ability to support fibroblasts and antibacterial properties.

## 2. Materials and Methods

### 2.1. Collection and Preparation of Trichopteran Silk

Caddisfly silk fiber produced by *H. angustipennis* larvae was obtained from pupal cocoons and used as short threads. Individuals collected from the natural environment were bred for 2 weeks under laboratory conditions (water temperature maintained at 14 °C, oxygen saturation at 4.5 mg/dm^3^) until they produced cocoons. Before they were placed in the laboratory setup, each larva was repeatedly rinsed with deionized water and deprived of food to empty their alimentary tracts and to eliminate contamination of the cocoons by metabolic products. Starvation lasted for 3 days, then the larvae were rinsed with deionized water again, the water in the containers was replaced and laboratory breeding was started. Before they were placed, in the laboratory setup, each larva was repeatedly rinsed with deionized water and deprived of food to empty their alimentary tracts. During the breeding process, water was exchanged every second day [28]. The cocoons obtained were mechanically rinsed in deionized water and then homogenized in a water solution using an X-120 knife homogenizer (CAT Ingenieurbüro GmbH, Ballrechten-Dottingen, Germany). Homogenization was performed at 3500 rpm for 4 min. Homogenates were steam sterilized by autoclaving at 121 °C and a pressure of 115 kPa for 20 min [28]. The silk prepared using this protocol was then freeze-dried.

According to the Polish regulations for field and laboratory studies, ethical approval was not required, as well as water tenant permission. The studied species is not protected by law in Poland and is listed in Annex II of the European Habitats and Species Directive.

### 2.2. Polycaprolactone (PCL) Foam Scaffolds Preparation

In the study, we used polycaprolactone (PCL) Capa 6800^®^ (Perstorp, Malmö, Sweden) and 1,4-dioxane (Chempur, Piekary Śląskie, Poland). PCL and PCL with 30 wt.% silk (PCL foams/modified silk) were prepared using the Thermally Induced Phase Separation Technique (TIPS) according to the procedure described in our previous study [40]. Briefly, a 5 wt.% solution in 1,4-dioxane was prepared using a magnetic stirrer (40 °C, 24 h). Next, silk was added to the PCL solution and mixed overnight (hybridization). Then, the mixture was frozen (−40 °C, 24 h) and freeze-dried (20 Pa, 24 h).

### 2.3. Differential Scanning Calorimetry (DSC)

Differential scanning calorimetry (DSC) analyses were carried out using a DSC1 instrument (Mettler Toledo, Greifensee, Switzerland) equipped with a Huber TC 100 intracooler as the cooling system. The measurements were performed over a temperature range from −70 °C to 100 °C, employing heating and cooling rates of 10 °C/min under a nitrogen atmosphere with a flow rate of 60 mL/min. The sample mass was approximately 3 mg. The resulting DSC curves were normalized to the sample weight and analyzed using STARe software, version 16.2 (Mettler Toledo, Switzerland).

The materials’ initial degree of crystallinity X_c_ was determined using equation X based on the first DSC heating scan.

(1) $$X_{c}=\frac{{∆H}_{m}}{{w∆H}_{m}^{100\%}}$$

where ΔH_m_—melting enthalpy of PCL [J/g], ΔH_m_^100%^—melting enthalpy of 100% crystalline PCL—135 J/g [41], w—PCL weight fraction in composite.

### 2.4. Thermogravimetric Analysis (TGA)

Thermogravimetric analysis (TGA) was performed using a TGA/DSC1 (Mettler Toledo, Greifensee, Switzerland). Measurements were conducted at a heating rate of 10 °C/min within a temperature range of 25–900 °C, under a constant nitrogen flow of 60 mL/min. The initial mass of each sample was approximately 3 mg.

### 2.5. Attenuated Total Reflectance—Fourier Transform Infrared Reflectance (ATR-FTIR)

ATR-FTIR spectra were recorded in ATR mode using a Nicolet iZ10 spectrometer (Thermo Scientific, Waltham, MA, USA) over the spectral range of 600–4000 cm^−1^. Each spectrum was acquired by averaging 32 scans at a resolution of 4 cm^−1^.

### 2.6. Water Contact Angle Measurement

Water contact angle measurements were performed using a PG-X contact angle goniometer (Testing Machines, New Castle, DE, USA). For each sample, a minimum of five measurements was taken, and the mean value along with the standard deviation was subsequently calculated.

### 2.7. Scanning Electron Microscope (SEM) Analysis

The morphology of the PCL foams and the PCL foams/modified silk was examined with an electron scanning microscope (Phenom ProX, Thermo Fisher, Waltham, MA, USA). The samples were examined using an accelerating voltage of 10 kV, with magnifications varying between 275× and 910×. Imaging was conducted using a charge reduction sample holder; thus, coating the samples with gold was not necessary. The pore size, namely two cross sections (crs1 and crs2) and circumference (L), was measured based on available SEM images using Leica LAS X software LASX_Office_1.4.3_26413 (Leica Microsystems GmbH, Frankfurt, Germany). Data are presented as mean ± SEM (standard error of the mean) of three separate experiments (three replicates for each assay).

### 2.8. Sterilization and Sample Preparation

Before biological testing, all composite samples were sterilized by gamma irradiation (35 kGy, ^60^Co source) at the Institute of Applied Radiation Chemistry, Technical University of Lodz (Lodz, Poland), following the procedure described previously [42]. For the cytotoxicity assays, the composites were cut into pieces corresponding to one-tenth of the well surface area, in accordance with ISO 10993-5:2009 [43]. For the evaluation of cell proliferation and adhesion via confocal microscopy, the composites were prepared as discs with a diameter of 7 mm.

### 2.9. Cell Culture and Expansion

The L929 (CCL-1) mouse fibroblast cell line was sourced from the American Type Culture Collection (ATCC, Manassas, VA, USA). Prior to experiments, fibroblasts were cultured in Roswell Park Memorial Institute (RPMI)-1640 medium supplemented with 10% heat-inactivated fetal bovine serum (FBS; HyClone Cytiva, Marlborough, MA, USA), penicillin (100 U/mL), and streptomycin (100 µg/mL) (Sigma Aldrich, Darmstadt, Germany) at 37 °C in a humidified atmosphere containing 5% CO_2_. Upon reaching confluence, cell monolayers were detached from the culture vessels using a 0.5% trypsin-EDTA solution (Gibco, Waltham, MA, USA) and resuspended in fresh culture medium. Cell viability and density were assessed using a Bruker chamber (Blaubrand, Wertheim, Germany) and the trypan blue exclusion assay, respectively. Only cell suspensions with a viability greater than 95% were employed in cytocompatibility studies. Morphological evaluation of the cell cultures was conducted using an inverted microscope (Motic AE2000, Xiamen, China).

The THP1-Blue NF-κB human monocytic cell line (InvivoGen, San Diego, CA, USA) was cultured in RPMI medium supplemented with 10% FBS, 25 mM 4-(2-hydroxyethyl)-1-piperazineethanesulfonic acid (HEPES), 100 U/mL penicillin, 100 μg/mL streptomycin, 2 mM glutamine, and selective agents (100 μg/mL normocin and 10 μg/mL blasticidin). Cells were maintained at 37 °C in a humidified incubator with 5% CO_2_. Cells at passages 4–6 were used for all experiments to ensure consistency and maintain cellular characteristics.

### 2.10. Direct Contact Cytotoxicity Assay

The metabolic activity of cells exposed to the test biomaterials was assessed using the 3-(4,5-dimethylthiazol-2-yl)-2,5-diphenyltetrazolium bromide (MTT) reduction assay, as previously described [44]. A cell suspension of 2 × 10^5^ L929 cells/mL was seeded (100 µL/well) into 96-well culture plates (Nunclon Delta Surface, Nunc, Rochester, NY, USA) and incubated overnight (37 °C, 5% CO_2_) to allow formation of confluent monolayers. Composites were then cut into pieces representing one-tenth of the well surface area and added to the cell monolayers in six replicates. Following an overnight incubation, 20 µL of MTT (Sigma Aldrich, Darmstadt, Germany) was added to each well, and incubation was continued for an additional 4 h. The supernatants were then discarded and replaced with 200 µL of dimethyl sulfoxide (DMSO). Absorbance was measured at 570 nm using a Multiskan EX reader (Thermo Scientific, Waltham, MA, USA). Cell cultures without any added biomaterials served as the non-treated control (NTC), while cultures treated with 2% hydrogen peroxide were used as the treated control (TC). Tubing samples from the Blood Collection Set (Vacutainer, BD, Franklin Lakes, NJ, USA) were used as the reference material.

### 2.11. Cell Proliferation Assay

The proliferation of L929 fibroblasts was assessed after 24, 48, 72, and 96 h of incubation with PCL foam scaffolds using the CyQuant Cell Proliferation Assay (Invitrogen, Thermo Fisher Scientific, Waltham, MA, USA), as previously described [45]. At the designated time points, cells embedded within the foam composites were washed with PBS and then stored at −80 °C. Prior to DNA quantification, the samples were thawed at room temperature, and the cells were lysed for 5 min in a buffer containing the CyQuant-GR dye, which specifically stains cellular DNA. Fluorescence was measured with an emission wavelength of 520 nm and an excitation wavelength of 480 nm using a SpectraMax^®^ i3x Multi-Mode Microplate Reader (Molecular Devices, San Jose, CA, USA). Standard curves were constructed using serial dilutions of known cell suspensions.

### 2.12. Visualization of Cell Adhesion and Penetration

After 96 h of incubation with L929 fibroblasts, the caddisfly silk, PCL foams, and the PCL foams/modified silk were washed with phosphate-buffered saline (PBS). Control samples consisted of cells cultured directly on microscope slides, which allowed for consistent imaging conditions and served as a reference for experimental groups. The cells were fixed with 3.7% paraformaldehyde (Sigma Aldrich, Saint Louis, MO, USA) for 30 min at room temperature. Subsequently, DNA was stained with DAPI (Thermo Fisher Scientific, Waltham, MA, USA) for 5 min (0.5 mg/mL) and Texas Red™ Phalloidin (Thermo Fisher Scientific, Waltham, MA, USA) for F-actin staining (0.1 μg/mL). Confocal images were captured using a TCS SP8 laser scanning microscope (Leica Microsystems, Frankfurt, Germany) equipped with a 63×/1.40 objective lens (HC PL APO CS2, Leica Microsystems, Frankfurt, Germany). Nuclei stained with DAPI were visualized with excitation at 405 nm and emission between 460 and 480 nm. F-actin staining with Texas Red™ Phalloidin was observed using a supercontinuum laser with excitation at 595 nm and emission wavelengths ranging from 610 to 630 nm. Image analysis and 3D reconstructions were performed using the Leica Application Suite X software LASX_Office_1.4.3_26413 (LAS X, Leica Microsystems, Frankfurt, Germany). The average cell size was calculated as the mean ± standard deviation (SD) from no fewer than ten cells across three independent fields of view.

### 2.13. Immunomodulatory Properties

The activation of the NF-κB signaling pathway was assessed using THP1-Blue™ NF-κB cells, as previously described [46]. Briefly, THP1-Blue™ NF-κB cells were seeded at a density of 1 × 10^5^ cells per well. Composites, cut into pieces representing one-tenth of the well surface area, were added to the selected wells (in six replicates) and incubated overnight at 37 °C in a humidified 5% CO_2_ atmosphere. Monocytes cultured in medium without additives served as the non-treated control (NTC), while those stimulated with LPS from *Escherichia coli* (100 ng/mL) were used as the treated control (TC) for NF-κB activation. Additionally, pre-incubation of composites with polymyxin B (PMB; 1 µg/mL, 30 min at 37 °C) was performed to distinguish endotoxin-dependent from non-endotoxin-related cell activation. The secreted embryonic alkaline phosphatase (SEAP) was quantified by mixing 20 μL of cell-free supernatant with 180 μL of QUANTI-Blue™ buffer (InvivoGen, San Diego, CA, USA), followed by incubation at 37 °C with 5% CO_2_ for 4 h. The optical density was measured at 650 nm using a Multiskan EX reader (Thermo Scientific, Waltham, MA, USA).

### 2.14. Resazurin Assay for Assessment of Antimicrobial Properties

The metabolic activity of bacterial cells exposed to the biomaterials was assessed by resazurin reduction, following the procedure outlined previously [47]. A 100 µL of bacterial suspensions of *Escherichia coli* ATTC 12287 (3.6 × 10^5^ CFU/mL) or *Staphylococcus aureus* (5.6 × 10^5^ CFU/mL) were transferred into 96-well black culture plates (Nunclon Delta Surface Black, Nunc, Rochester, NY, USA) and incubated for 30 min (37 °C). The composites were then cut into pieces representing one-tenth of the well surface area and placed into the corresponding wells (in six replicates). Following overnight incubation, 20 µL of a 0.02% resazurin solution (sterilized through 0.22 µm filters) was added to each well to assess cell viability, and the incubation was extended for an additional 4 h. During this time, the non-fluorescent blue dye resazurin is able to penetrate the cell, where it is transformed into a pink fluorescent dye, resorufin, in response to the activity of reductases present in the cell. Therefore, this process can only occur in living cells. After 4 h of incubation at 37 °C, fluorescence was measured using a SpectraMax^®^ i3x Multi-Mode Microplate Reader (Molecular Devices, San Jose, CA, USA) at excitation and emission wavelengths of λex = 530 nm and λem = 590 nm, respectively. Cell viability was calculated relative to the non-treated control exposed to the tested biomaterials, which was assigned a metabolic activity value of 100%.

### 2.15. Statistical Analysis

The statistical analysis was performed using GraphPad Prism 6 (GraphPad Software, San Diego, CA, USA). The normality of the data was evaluated using the Kolmogorov–Smirnov test. Intra-group comparisons were made using the non-parametric Mann–Whitney U test. Some data were analyzed using a regular one-way ANOVA followed by Tukey’s multiple comparisons. The differences were considered significant when a *p*-value was <0.05.

## 3. Results

This study aimed to evaluate the potential of caddisfly silk (*Hydropsyche angustipennis*) as a bioactive modifier for PCL foams in tissue engineering applications. By combining the natural properties of insect silk with a widely used synthetic polymer, we sought to enhance the physicochemical and biological performance of the resulting biocomposites. Here, we present the key findings related to the structural characteristics, cytocompatibility, and bioactivity of the PCL foams/modified silk foams. Emphasis is placed on the improvements in fibroblast attachment and proliferation, the activation of the NF-κB signaling pathway, and the maintenance of antimicrobial activity of PCL foams/modified silk compared to unmodified PCL foam.

### 3.1. Biomaterials Properties

The physicochemical properties of biomaterials are responsible for maintaining an optimal environment, ensuring the functional activity of cells and tissue regeneration.

**Figure 1:**
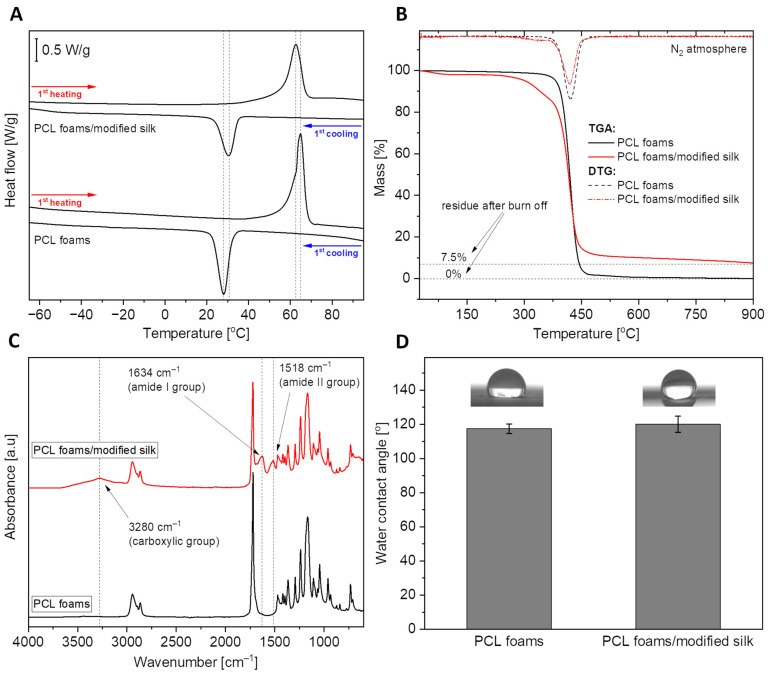
Physicochemical characteristics of PCL foams and PCL foams/modified silk scaffolds: (**A**). First heating/cooling DSC curves, (**B**) thermogravimetric curves with their first derivatives (DTG), (**C**) normalized FTIR spectra; dashed lines indicating distinct bands originating from the silk fraction, and (**D**) comparison of water contact angle values.

Figure 1A shows the curves from the first heating and first cooling scans for both pure polycaprolactone and composite material (PCL foams/modified silk). The courses of the corresponding curves are very similar for each sample. A clear endothermic effect related to the polymer melting process can be observed during the heating of materials. The observed effect was characterized by two parameters: T_m_—melting point and ΔH_m_—melting enthalpy, the values of which are listed in Table 1. Despite the significant addition of silk fibers (30% by weight), the melting point changed slightly from 64.9 °C to 63.0 °C. Based on the enthalpy of melting, the degree of crystallinity of the polymer was calculated for both materials. It turns out that the PCL foams/modified silk material has a higher degree of crystallinity by about 9%. This proves the nucleating properties of silk fibers. The second effect, exothermic, visible on the cooling scans, demonstrates the crystallization of the polymer from the melt during its cooling process. This effect was also characterized by two parameters: T_c_—temperature of crystallization and ΔH_c_—enthalpy of crystallization. The enthalpy values given in the table have been normalized to the weight of the sample. However, by normalizing to the weight of the polymer, a value of 57.2 J/g is obtained for the composite foam, which is higher than that of neat PCL (54.8 J/g). The increased value of ΔH_c_, together with the increased crystallization temperature, confirms the nucleation effect after introducing the filler into the system.

Figure 1B presents the thermogravimetric curves of the materials, demonstarting their thermal stability. To quantify them, three parameters were determined for both curves: T_(−5%)_, the temperature at a loss of 5% of the sample weight; T_(DTG)_, the temperature corresponding to the peak maximum of the first derivative of the TGA curve, indicating the most rapid loss of sample weight; and Residue, the amount of material remaining at 900 °C. These parameters are listed in Table 1. First, the material based on neat PCL is characterized by high thermal stability, showing rapid weight loss above 350 °C. Only at over 370 °C is there a loss of 5% of the sample weight. The situation is different in the case of PCL foams/modified silk material. The composite is less stable, and mass loss occurs in several stages, which is also clearly visible during the first derivative curve. The first weight loss occurs already in the temperature range of 25–100 °C. Then, just above 300 °C, the second stage of weight loss occurs, directly related to the degradation of caddisfly fibers. The highest mass loss rate at this stage occurs at a temperature of approximately 325 °C. The final, largest mass loss reaches its maximum rate at about 410 °C and is directly related to the degradation of the PCL. After all, although PCL foam degrades completely at 900 °C, approximately 7.5% residue is observable with PCL foams/modified silk.

The FTIR analysis allowed for qualitative confirmation of the chemical composition of PCL foams and PCL foams/modified silk. The course of the black curve visible in Figure 1C corresponds to that of pure PCL. On the other hand, in the spectrum recorded for the PCL foams/modified silk, additional distinct bands were observed, indicating the presence of caddisfly fibers in the system. These bands show the presence of carboxyl, amide I, and amide II groups in the fiber structure, confirming the protein-based structure of silk fibers. The presence of a significant number of polar groups and the ability to absorb water resulting from the TGA tests, suggests an improvement in the wettability of the composite material compared to the neat polymer. However, as shown in Figure 1D, the wettability of the surface with water practically did not change.

### 3.2. Microstructure of PCL Foams/Modified Silk

**Figure 2:**
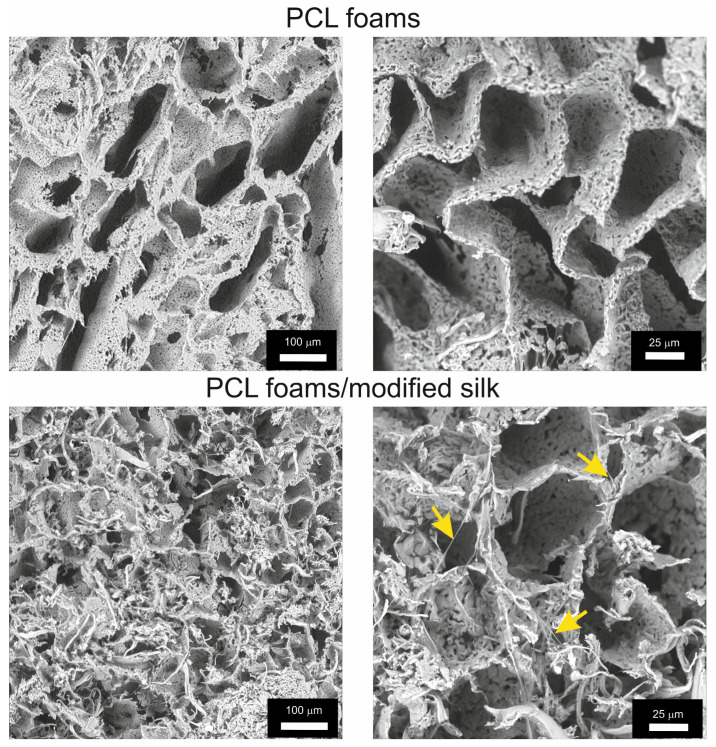
The microstructure of PCL foams and PCL foams/modified silk. Silk fibers are marked with yellow arrows.

The PCL foams characterized a porous structure, while the introduction of caddisfly silk into the structure of the PCL foams/modified silk resulted in the deposition of silk in the free spaces, where fragments of fibers were visible inside the pores (Figure 2).

The pore size in both PCL foams and PCL/modified silk foams was characterized using three measurements: two cross-sections (crs1 and crs2) and the circumference.

In PCL foams, the minimum and maximum recorded lengths for crs1 were 24 μm and 264 μm, respectively, with a mean value of 97 ± 55 μm. For crs2, the measurements were 17 μm (minimum), 53 μm (maximum), and a mean of 22 ± 26 μm. The circumference measurements ranged from 74 μm to 633 μm, with a mean value of 264 ± 136 μm.

The measurements for PCL foams/modified silk were lower. The mean value for crs1 was 66 ± 16 μm, with a minimum and maximum recorded lengths of 45 μm and 117 μm, respectively. For crs2, the measurements were as follows: 30 μm (minimum), 312 μm (maximum), and mean = 43 ± 9 μm. The circumference ranged from 140 μm to 312 μm, with a mean value of 190 ± 43 μm.

### 3.3. Cell Viability and Cell Proliferation

**Figure 3:**
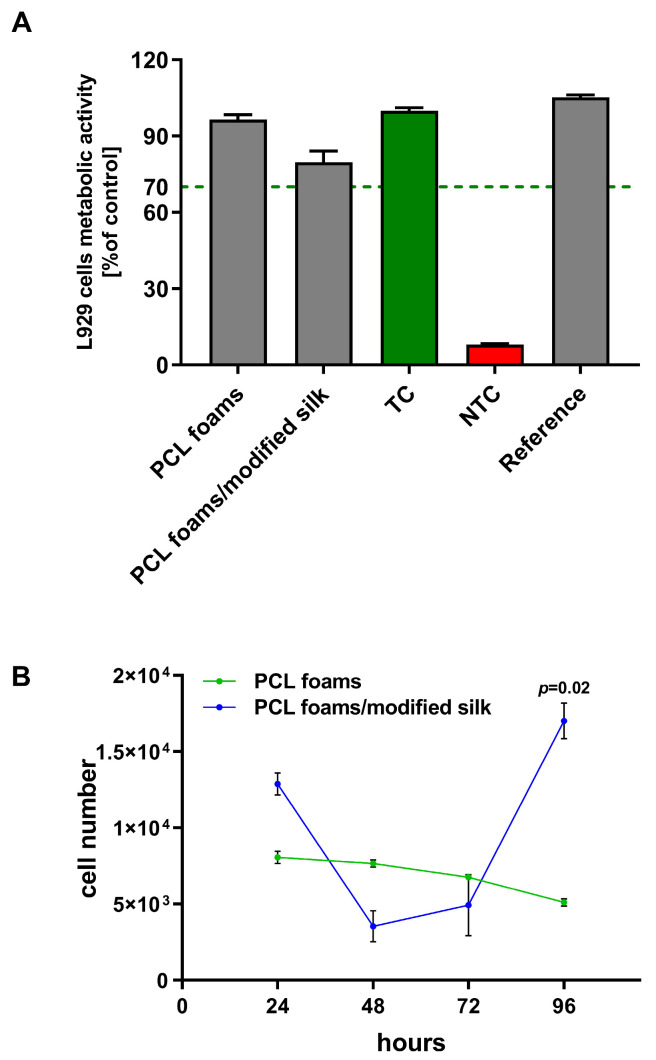
(**A**) The viability of L929 fibroblasts after 24 h of incubation in the milieu of obtained foam scaffolds. Data are presented as mean ± SEM (standard error of the mean) of three separate experiments (six replicates for each assay). The dashed line indicates the minimum level (70%) of the cells’ metabolic activity required to recognize the biomaterial as noncytotoxic at the in vitro level. TC—treated control (red bar) and NTC—non-treated control (green bar), in terms of cell metabolic activity, according to ISO 10993-5:2009 and our previous research [43]; (**A**) demonstrates that both PCL foams and PCL foams/modified silk supported high fibroblast viability after 24 h, meeting ISO 10993-5 cytocompatibility criteria. (**B**) The proliferation of L929 fibroblasts in the PCL and PCL foams/modified silk was monitored for up to 96 h of culture. The results are shown as mean ± SEM (standard error of the mean) from three separate experiments. (**B**) shows that PCL foams/modified silk significantly enhanced L929 fibroblast proliferation over 96 h compared to unmodified PCL foams, indicating their potential to support cell growth in tissue regeneration.

Cytocompatibility is considered to be one of the most important requirements of materials used in biomedical applications. Thus, the 24 h MTT reduction assay was performed to evaluate the initial cellular attachment and metabolic activity of L929 fibroblasts incubated with the biocomposites, in accordance with ISO 10993-5:2009 [43] recommendations, rather than to assess long-term viability. As shown in Figure 3A, the viability of murine fibroblasts exposed to PCL foams and PCL foams/modified silk reached 96.6% ± 1.9% and 79.8% ± 4.3%, respectively.

A proliferation assay was used in this study to measure the relative DNA content and evaluate the number of L929 cells within PCL and PCL foams/modified silk for up to 96 h of culture (Figure 3B). The number of L929 fibroblasts colonizing the PCL foams decreased slowly with the duration of the experiment from 7.7 × 10^3^ ± 0.1 × 10^3^ cells at 48 h to 5.1 × 10^4^ ± 0.1 × 10^4^ cells at 96 h. Interestingly, the number of cells detected on the PCL foams/modified silk decreased in the initial stages of colonization (up to 48 h: 3.5 × 10^4^ ± 1.0 × 10^4^) and then increased rapidly, reaching 17.0 × 10^4^ ± 1.2 × 10^4^ at 96 h, which was statistically significant (*p* = 0.02) as compared to the number of cells colonizing the PCL foams.

### 3.4. Cell Attachment and Penetration

**Figure 4:**
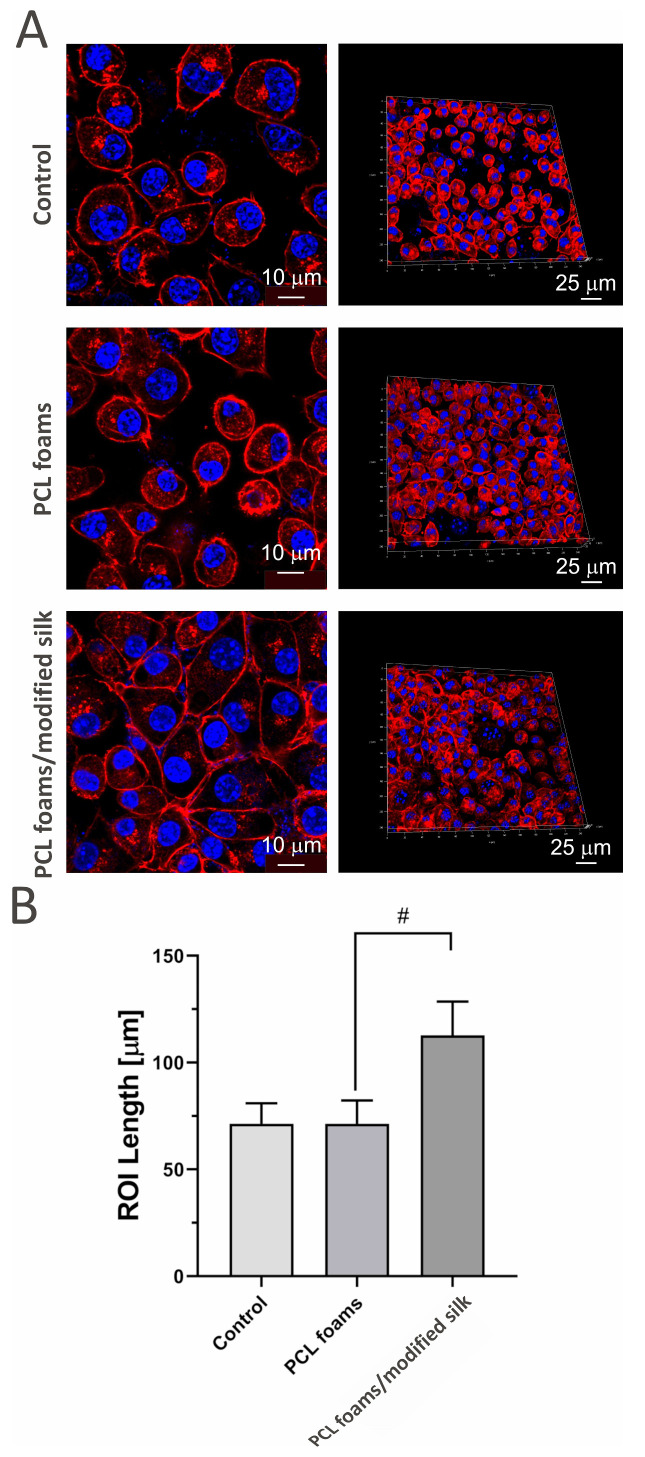
The morphology (**A**) and average size (**B**) of L929 (mouse skin fibroblast cells) after 96 h of incubation with tested PCL foams and PCL foams/modified silk, as compared to control cells grown directly on microscope slides. Confocal microscopy visualized cellular structures using DAPI (nuclei; blue) and Texas Red™ Phalloidin (F-actin filaments; red) staining. Scale bars indicate 10 µm (2D images) and 25 µm (3D reconstructions). The results represent the mean values ± SEM of three separate experiments. # *p* < 0.05 between the PCL and PCL foams/modified silk, based on the results of a one-way ANOVA (Kruskal–Wallis test) evaluation. The figure shows that PCL foams/modified silk enhanced fibroblast adhesion and altered cell morphology, promoting more elongated and spread cells compared to PCL foams and control surfaces.

To investigate the impact of PCL foams and PCL foams/modified silk after 96 h of incubation on cell morphology and penetration, L929 mouse skin fibroblasts were cultured for 96 h on three types of surfaces: microscope slides (control), pure PCL foams, and PCL foams/modified silk. After incubation, samples were washed, fixed, and stained for confocal microscopy. As shown in Figure 4**,** the PCL foams/modified silk supported enhanced adhesion of murine fibroblasts, forming a more compact monolayer compared to control cells. Cells seeded on PCL foams exhibited regular morphology and physiological nuclei, comparable to the control group. However, modification with caddisfly silk significantly influenced cell morphology by increasing the length and circumference of the cells.

### 3.5. Antimicrobial Properties

**Figure 5:**
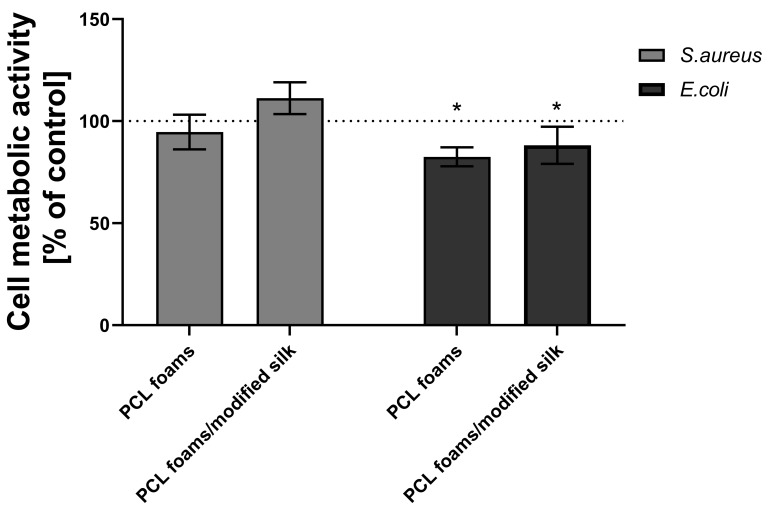
The antimicrobial properties after 24 h incubation with tested compounds (PCL foams and PCL foams/modified silk) against *S. aureus* or *E. coli*. The results represent the mean values ± SEM of three separate experiments. * *p* < 0.05 of the PCL and PCL-modified composites in relation to non-treated samples (100% viability of bacterial cells), based on the results of a one-way ANOVA (Kruskal–Wallis test) evaluation. The figure shows that both PCL foams and PCL foams/modified silk reduced *E. coli* metabolic activity, while no significant effect was observed against *S. aureus* after 24 h.

The antimicrobial activity of PCL foams and PCL foams/modified silk after 24 h of incubation with *S. aureus* or *E. coli* was determined by the resazurin reduction tests. After 24 h of incubation with *S. aureus*, the metabolic activity of bacteria exposed to PCL foams (94.7% ± 8.5%) and PCL foams/modified silk (111.2% ± 7.8%) remained comparable to the untreated control group (Figure 5). Test samples incubated with *E. coli* caused a statistically significant decrease in the metabolic activity of the cells after 24 h of incubation. In the case of *E. coli*, both PCL foams (82.5% ± 9.3%, *p* < 0.05) and PCL foams/modified silk (88.1% ± 4.7%, *p* < 0.05) caused a statistically significant reduction in bacterial metabolic activity after 24 h of incubation; thus, the addition of caddisfly silk did not alter the antimicrobial activity of PCL.

### 3.6. Immunostimulatory Activity

**Figure 6:**
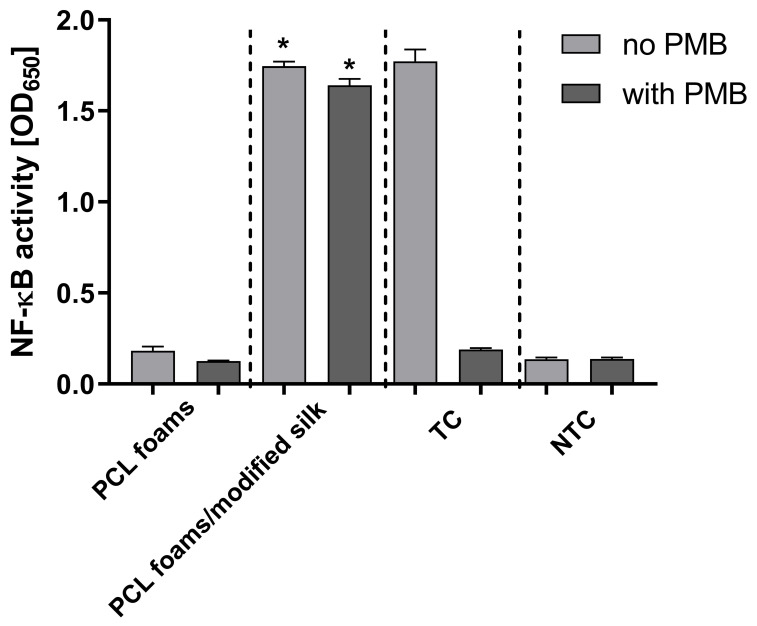
The activation of the NF-κB transcription factor in THP1-Blue NF-κB monocytes incubated for 24 h with tested PCL foams and PCL foams/modified silk in comparison with monocytes stimulated with lipopolysaccharide (LPS) of *E. coli* (TC) or cell culture medium (NTC). In parallel, samples were preincubated with polymyxin B (PMB). Data are presented as mean ± SEM (standard error of the mean) of three separate experiments (six replicates of each assay). Data were compared using a regular one-way ANOVA followed by Tukey’s multiple comparisons. * *p* < 0.05 of the PCL foams/modified silk in relation to PCL foams. The figure shows that PCL foams/modified silk significantly activated the NF-κB pathway in THP1-NF-κB reported monocytes, suggesting their potential to modulate inflammatory responses.

We used THP1-Blue NF-κB human monocytes, carrying an NF-κB-inducible SEAP reporter construct, to explore the potential activation of the NF-κB signal transduction pathway via tested PCL materials. The PCL foams/modified silk induced NF-κB activation up to the level induced by LPS *E. coli* (treated control), and it was significantly higher (*p* < 0.05) as compared to PCL foams (Figure 6). However, the addition of polymyxin B (PMB), an inhibitor of LPS, had no effect on PCL foams/modified silk scaffolds-induced changes in the secretion of embryonic alkaline phosphatase (SEAP) but completely inhibited the LPS-induced increases in SEAP production.

## 4. Discussion

Biomedical engineering integrates materials science with biomedical knowledge to design and develop innovative biomaterials tailored for tissue regeneration. Among the wide array of materials explored, silk-modified biomaterials have gained increasing attention due to their mechanical strength, biocompatibility, and biodegradability. Silk-based composites are being extensively studied and applied across a broad spectrum of biomedical fields, including bone, cartilage, and skin tissue regeneration, as well as in drug delivery systems [48].

This study presents the development of PCL-based biomaterials modified with caddisfly silk and evaluates their biological properties, including cytocompatibility, support for fibroblast proliferation and attachment, activation of the NF-κB signaling pathway in a reporter monocyte model, and antibacterial activity.

In our study, we demonstrated that the integration of natural silk obtained from caddisflies into synthetic PCL foams modified their physicochemical properties. The increased crystallinity observed in PCL foams/modified silk confirmed the nucleating effect of silk fibers, aligning with earlier reports in the literature [40]; however, the significant addition of silk to the PCL only slightly affected the melting point. It is worth emphasizing that the low melting point of the developed PCL foams/modified silk (T_m_ = 63 °C) results in favorable processing characteristics, combined with high thermal stability. This enables the potential fabrication of such materials via solvent-free thermal processing techniques, such as extrusion, injection molding, or various 3D printing methods.

The differences in thermal stability at 100 °C observed in our study between PCL foams and PCL foams/modified silk may be related to the strong ability of silk fibers to absorb water or other polar solvents and the subsequent evaporation of this fraction [28]. Similarly, the increased amount of residue at 900 °C may be attributed to inorganic impurities present in the caddisfly fibers within the PCL foams/modified silk, which require much higher temperatures to decompose or evaporate [28,49].

FTIR analyses confirmed the consistency of the spectra for PCL foams with those reported in the literature [50], as well as the presence of caddisfly silk fibers in the PCL foams/modified silk. This was evidenced by the appearance of characteristic absorption bands (carboxyl, amide I, and amide II groups) in the fiber structure, which confirmed the protein-based nature of silk fibers [51]. The wettability of the PCL foams/modified silk surface with water did not change significantly compared to PCL foams. This is most likely related not so much to the surface’s chemical composition as to its morphology [52].

The surface topography of biocomposites plays a crucial role in regulating cell adhesion. Cell migration, attachment, and penetration of biomaterials depend on the micro- and macrotopography of the composite surface, including the size and distribution of pores [53]. In our study, we demonstrated that the incorporation of silk into PCL foams reduces the average pore size compared to unmodified PCL foams. Moreover, the presence of caddisfly silk may positively influence cell attachment and facilitate foam penetration.

Biomaterials designed for regenerative medicine must be highly cytocompatible and biocompatible to support cells proliferation and stable long-term integration with surrounding tissues [54]. Both PCL foams and PCL foams/modified silk met the ISO criterion of maintaining the viability of at least 70% of cells after incubation in the biomaterial’s milieu. Our results are in accordance with those of Farokhi et al. [55]. Liu et al. reached similar conclusions, showing that a silk fibroin-based biomaterial derived from silkworms was non-cytotoxic to L929 fibroblasts and did not negatively affect their cell cycle or apoptosis [56]. Moreover, the biosafety of SF has also been demonstrated in studies involving the breast cancer cell lines MCF-7 and MDA-MB-231 [57].

Additionally, PCL foams/modified silk stimulated L929 fibroblast proliferation. This observation is consistent with previous studies demonstrating that silk proteins support the growth and proliferation of fibroblasts and keratinocytes [58]. For example, silk sericin has been reported to enhance the proliferation and differentiation of keratinocytes involved in wound re-epithelialization, as well as fibroblasts and endothelial cells contributing to tissue regeneration [59]. Similarly, Moisenovich et al. [60] showed that silk fibroin scaffolds promote mouse embryonic fibroblast adhesion and proliferation, highlighting their potential in regenerative medicine applications. On the contrary, silk fibroin peptide (SFP) inhibited the proliferation of lung cancer cells (A549 and H460) in a dose-dependent manner and suppressed the clonogenic activity [61].

Silk proteins derived from various insect sources contain unique amino acids, that, when combined with synthetic polymers, lead to diverse surface chemistry, roughness, mechanical properties, and wettability. This often results in enhanced cell attachment and adhesion, ultimately promoting their spreading on the biocomposite [62]. In this study, the modification of PCL foams with caddisfly silk significantly altered cell morphology by increasing cell length and perimeter compared to unmodified PCL foams. This may enhance long-term colonization of the composites, although further studies are needed to confirm this. Previously, it was demonstrated that silk optimization enhances adhesion properties to various surfaces in aqueous environments [31,32,63]. The fibroblasts’ attachment to silk is comparably high compared to their adhesion to collagen films [3]. Additionally, our preliminary examination indicated increased growth of mouse fibroblasts (NIH/3T3) and human dermal fibroblast (BJ) cells, enhanced attachment, and proliferation on the caddisfly silk surface [28]. Zakeri Siavashani et al. reported that silk-based scaffolds supported fibroblast (MG-63) attachment on the biomaterial surface, provided physiological morphology of the cells, and enabled well-spread fibroblasts with visible cell-to-cell contact [64].

One of the desired properties of biocomposites intended for tissue regeneration is antibacterial activity, which can significantly reduce the risk of post-implantation infections. In our study, both PCL foams and PCL foams/modified silk reduced the metabolic activity of *E. coli*. Our findings are consistent with previously reported data [65,66]. Notably, the reduction in metabolic activity was similar for both test samples. Future research will focus on identifying pathogenic species for which PCL foams/modified silk may serve as a composite with antibacterial properties.

The successful integration of biocomposites with surrounding tissues largely depends on the material not triggering a strong inflammatory response. In this study, NF-κB activation was induced by PCL foams/modified silk, whereas no such activation was observed for unmodified PCL foams. The activation of the NF-κB is crucial for stimulating the secretion of pro-inflammatory cytokines such as tumor necrosis factor (TNF)-α, interleukin (IL)-6, and monocyte chemoattractant protein-1 (MCP-1), which promote proliferation and migration of fibroblasts and keratinocytes [67,68]. NF-κB-activating biomaterials can enhance cell growth and migration to the lesion site and positively affect the remodeling of damaged tissue [69,70]. Hence, further studies on PCL foams/modified silk regarding their pro-inflammatory properties for targeted tissue regeneration are required.

Despite obtaining promising results for our composites and the proposed use of caddisfly silk as a modifier for PCL foams, certain limitations remain. First and foremost, finding insect larvae at the appropriate developmental stage to obtain sufficient silk for large-scale production remains problematic. Furthermore, the laboratory cultivation of caddisflies and silk harvesting is still at an unsatisfactory level to develop more diversified versions of the PCL foams/modified silk composites.

The caddisfly silk could be used in medical applications for bone or skin tissue regeneration due to its molecular structure, strength, resistance to deformation, and bioactivity [28,48]. In the upcoming studies, we plan to research the mechanical properties and biodegradation, and further investigate the interaction of the PCL foams/modified silk with immune system cells. Additionally, the bioactivity of the foams will be tested in models of osteoblasts, osteocytes, and keratinocytes to assess their potential in regenerating bone and skin tissues.

## 5. Conclusions

In this study, a comparison between scaffolds based on pure PCL and PCL with the addition of 30% by weight of caddisfly silk fibers was conducted. The materials are characterized by distinct macroporosity, which is disrupted by the presence of the fibrous filler. Composite foams exhibit greater water absorption and lower thermal stability compared to the pure polymeric ones. Nevertheless, in both cases, the thermal stability of the materials exceeds 290 °C. This, combined with the low melting temperature (ranging from 63 °C to 65 °C), enables the potentially effective processing of materials using thermal methods, such as material extrusion 3D printing or injection molding. Moreover, the addition of fibers did not significantly affect the surface wettability, which remains around 120 degrees in both cases. The investigated PCL foams/modified silk were found to be fully cytocompatible. Furthermore, the caddisfly silk surface of foams promoted a more effective adhesion and proliferation of fibroblasts L929 compared to the PCL matrix. It is worth noting that the tested PCL foams/modified silk demonstrated the induction of NF-κB while excluding bacterial LPS-mediated stimulation measured in the monocyte/macrophage inflammation sensing system using human THP1-Blue NF-κB cells. Additionally, foams modified with caddisfly silk, as well as PCL, showed antimicrobial activity only against *E. coli*. Overall, the biological assessment of the PCL foams/modified silk makes them promising candidates suitable for medical or dental applications.

## 6. Patents

The results of the work described in this article resulted in the receipt of a Polish Patent No. PL.244676/2024.

## Figures and Tables

**Table 1:** Physicochemical parameters of the tested samples.

Sample	ΔH_m_ [J/g]	T_m_ [°C]	ΔH_c_ [J/g]	T_c_ [°C]	X_c_ [%]	T_(−5%)_ [°C]	T_(DTG)_ [°C]	Residue [%]	WCA [°]
PCL foams	92.0	64.9	−54.8	28.2	68.1	373.6	412.8	0	117.4 ± 2.7
PCL foams/ modified silk	73.4	63.0	−40.7	30.6	77.7	291.0	411.0	7.5	120.0 ± 4.7

## Data Availability

The data generated during this study are available at the University of Lodz, Faculty of Biology and Environmental Protection, Department of Immunology and Infectious Biology, Lodz, 90-237, Poland, and they are available from the corresponding authors upon request.
